# Melanoma cells influence the differentiation pattern of human epidermal keratinocytes

**DOI:** 10.1186/1476-4598-14-1

**Published:** 2015-01-05

**Authors:** Ondřej Kodet, Lukáš Lacina, Eliška Krejčí, Barbora Dvořánková, Miloš Grim, Jiří Štork, Daniela Kodetová, Čestmír Vlček, Jana Šáchová, Michal Kolář, Hynek Strnad, Karel Smetana

**Affiliations:** 1st Faculty of Medicine, Institute of Anatomy, Charles University in Prague, U Nemocnice 3, CZ-12800 Prague, Czech Republic; Department of Dermatovenerology, Charles University in Prague, 1st Faculty of Medicine and General University Hospital, U Nemocnice 2, CZ-12800 Prague, Czech Republic; Institute of Medical Biology, A*STAR, 8A Biomedical Grove, No. 06-06 Immunos, Singapore, 138648 Singapore; 2nd Faculty of Medicine, Institute of Pathology and Molecular Medicine, Charles University in Prague, V Úvalu 84, CZ-15006 Prague, Czech Republic; Institute of Molecular Genetics, Academy of Sciences of the Czech Republic, Laboratory of Genomics and Bioinformatics, Vídeňská 1083, CZ-14220 Prague, Czech Republic

**Keywords:** Melanoma, Cancer microenvironment, Melanocyte, Intercellular interaction, Pseudoepitheliomatous hyperplasia

## Abstract

**Background:**

Nodular melanoma is one of the most life threatening tumors with still poor therapeutic outcome. Similarly to other tumors, permissive microenvironment is essential for melanoma progression. Features of this microenvironment are arising from molecular crosstalk between the melanoma cells (MC) and the surrounding cell populations in the context of skin tissue. Here, we study the effect of melanoma cells on human primary keratinocytes (HPK). Presence of MC is as an important modulator of the tumor microenvironment and we compare it to the effect of nonmalignant lowly differentiated cells also originating from neural crest (NCSC).

**Methods:**

Comparative morphometrical and immunohistochemical analysis of epidermis surrounding nodular melanoma (n = 100) was performed. Data were compared to results of transcriptome profiling of *in vitro* models, in which HPK were co-cultured with MC, normal human melanocytes, and NCSC, respectively. Differentially expressed candidate genes were verified by RT-qPCR. Biological activity of candidate proteins was assessed on cultured HPK.

**Results:**

Epidermis surrounding nodular melanoma exhibits hyperplastic features in 90% of cases. This hyperplastic region exhibits aberrant suprabasal expression of keratin 14 accompanied by loss of keratin 10. We observe that MC and NCSC are able to increase expression of keratins 8, 14, 19, and vimentin in the co-cultured HPK. This in vitro finding partially correlates with pseudoepitheliomatous hyperplasia observed in melanoma biopsies. We provide evidence of FGF-2, CXCL-1, IL-8, and VEGF-A participation in the activity of melanoma cells on keratinocytes.

**Conclusion:**

We conclude that the MC are able to influence locally the differentiation pattern of keratinocytes *in vivo* as well as *in vitro.* This interaction further highlights the role of intercellular interactions in melanoma. The reciprocal role of activated keratinocytes on biology of melanoma cells shall be verified in the future.

**Electronic supplementary material:**

The online version of this article (doi:10.1186/1476-4598-14-1) contains supplementary material, which is available to authorized users.

## Background

Malignant melanoma is an aggressive tumor with increasing incidence and still limited therapeutic outcome in advanced disease. Permissive microenvironment is an essential condition for growth of malignant cells, including the melanoma cells, in the organism [1, 2]. Various non-malignant cell types present in tumor stroma collectively contribute to formation of the a specific milieu for cancer stem cells [3]. The stroma of tumors is usually composed of fibroblasts, producing not only extracellular matrix but also plethora of important cytokines, immune cells and endothelial cells of capillaries. The stromal fibroblasts (also known as cancer-associated fibroblasts) are able to significantly influence the phenotype of co-cultured epithelial cells and to affect epithelial to mesenchymal transition in them [4, 5]. Mutual crosstalk between the stromal fibroblasts and the melanoma cells has been also described [6]. Similarly to the other types of tumors, extensive macrophage infiltration can stimulate aggressiveness of melanoma [7]. In case of melanoma progression, keratinocytes as a principal epidermal cell population must be also taken in account in these schemes.

Melanocytes are normally present in the basal layer of human epidermis. Their precursors, the neural crest-originated stem cells, occur in the bulge of outer root sheath of hair follicle in human adult skin [8]. Evidence of MC impact on the epidermal microenvironment was described and discussed previously, however the molecular mechanism of this interaction was poorly understood [9, 10]. In this study the effects of melanoma cells on ‘bystander’ epidermal cells (keratinocytes) and mediators of this activity are investigated. We demonstrate the effect of nodular melanoma on morphology and differentiation pattern of the epidermis in the vicinity of the tumor. The histopathological findings are correlated with *in vitro* effect of melanoma cells and their precursors (NCSC) on HPK.

## Methods

### Tissue samples and cell lines

All cells and tissues were obtained after approval of the local ethical committee with informed consent of the patients. Paraffin embedded samples of human nodular melanomas (n = 100) were used for morphometric and histological analysis. Immunohistochemical analysis was performed on 20 of them (data summarized in Table 1). Two frozen samples of intraepidermal naevi, 3 samples of melanoma metastasis to skin, and samples of normal breast skin were also employed.

**Table 1:** **Characterization of the group of 100 patients with nodular melanomas**

Characterisation				Localisation				
Sex	Group	Mean age (years)	Breslow index range/mean	Back	Upper limb	Lower limb	Trunk	Head & neck
**Male**	49	61	0.7-15/4.2	15	12	8	12	2
**Female**	51	54	0.8-8.8/3.3	15	17	9	6	4

Human primary keratinocytes were isolated from donor skin and cultured in keratinocyte growth medium as described by Strnad and coworkers [5]. Human neural crest-originated stem cells (NCSC) were isolated from human hair follicles and cultured with addition of 5% chicken embryonic extract (CEE) [11]. Immortalized keratinocyte cell line HaCaT [12] was purchased from vendor (Cell Line Service, Eppelheim, Germany). Mouse embryonic 3T3 fibroblasts used for feeder were obtained from Department of Burn Surgery, 3rd Faculty of Medicine, Charles University, Prague).

Highly metastatic BLM melanoma cell line was a gift from L. van Kempen and H. van Krieken (Department of Pathology, Radboud University, Nijmegen, the Netherlands), commercially available metastatic melanoma lines A2058 and G361 were purchased from ATCC® HTB-43™). Primary melanoma cells (ASC passage P1 and P3) were isolated by us from ascitic fluid obtained by alleviating abdominal paracentesis in stage IV melanoma patient with multiple liver metastases. Normal human neonatal highly pigmented melanocytes (HPM) were purchased from J. Vachtenheim (Institute of Medical Biochemistry and Laboratory Medicine 1st Faculty of Medicine, Charles University, Prague, Czech Republic) and maintained in serum free medium M254 with HMGS supplement (Gibco, USA). All cultures during the experiment were kept at 37°C and 5% CO_2_ in humidified incubator in DMEM with 10%FBS, if not indicated otherwise below.

### Tissue sections preparation

The tissue sections were prepared from paraffin embedded nodular melanoma samples and from Tissue-Tek (Sakkura, Zoeterwoude, Netherlands) embedded frozen samples of normal skin, intraepidermal naevi or melanoma cutaneous metastases.

### Cell culture, cell culture in a transwell system, for transcriptomics, RT-qPCR and proliferation in conditioned media

For co-culture studies, HPK were inoculated on Mitomycin C–treated 3T3 feeder cells at the density 30,000 cells/cm^2^ [13] in the lower chamber of a 6-well plate transwell system (BD Falcon, Franklin Lakes, NJ, USA). Next day, PET-inserts (pore size 1 μm) with proper cells (BLM, NCSC and HPM respectively) were added. Highly proliferating BLM cells were seeded at the density of 500 cells/cm^2^, NCSC and HPM at the density 5,000 cells/cm^2^. The cells were co-cultured for six days in keratinocyte medium [5]. Keratinocytes cultured on feeder alone were used as a control.

For transcriptome analysis, BLM cells, A2058, G361, primary cultures of ascitic melanoma cells (ASC P1 and P3), HPM and NCSC cultured until they reached 90% confluence. The cultures were then briefly washed with ice cold PBS and harvested in RLT lysis buffer (QIAGEN Inc., Valencia, CA, USA). To exclude the specific influence of culture media, cells were harvested from either DMEM with 10% FBS or α-MEM with 10% FBS and 5% of CEE.

For further RT-qPCR verification of candidate target genes, cell cultures were performed under identical conditions as above, but also in parallels with and without FBS.

The influence of BLM cells on keratinocyte proliferation was tested by cultivation of HaCaT cells (2,000 per well) in 96-well plate (MIDSCI, St. Louis, USA) over 96 hours. The analysis was performed employing the IncuCyte FLR instrument and software (Essen BioScience, Inc. AnnArbor, Michigan, USA) according to the protocol provided by the supplier.

Based on microarray and RT-qPCR analysis, we selected upregulated genes that can exert the biological activity of melanoma cells on keratinocytes. Their effects were tested with substances purchased from RD system (USA) using enrichment by IL-8 (2.5 ng/ml), CXCL-1 (0.04 ng/ml), FGF-2 (10 ng/ml), and VEGF-A (0.4 ng/ml) [14, 15].

### Immunohistochemistry and immunofluorescence

The panel of antigens was visualized by immunohistochemistry and immunocytochemistry (Additional file 1: Table S1). The nuclei were counterstained by 4′,6-diamidino-2-phenylindole (DAPI; Sigma-Aldrich, Prague, Czech Republic). The control of the specificity of the reaction was performed with isotype control of the same species. Imaging was performed on Eclipse 90i fluorescence microscope (Nikon, Prague, Czech Republic) equipped with a Cool-1300Q CCD camera (Vosskühler, Osnabrück, Germany) and the computer-assisted image analysis system LUCIA 5.1 (Laboratory Imaging, Prague, Czech Republic).

### Transcriptome analysis

Total RNA was isolated using RNeasy Micro Kit (Qiagen, MD, USA). RNA integrity was analyzed by Agilent Bioanalyzer 2100 (Agilent). Only the samples with intact RNA profiles were used for expression profiling (RIN > 9).

Illumina HumanHT-12 v4 Expression BeadChips (Illumina, CA, USA) were used for the microarray analysis following the standard protocol. In brief, 200 ng RNA was amplified with Illumina TotalPrep RNA Amplification Kit (Ambion, TX, USA) and 750 ng of labeled RNA was hybridized on the chip according to the manufacture procedure. The analysis was performed in two biological replicates per group. The raw data were preprocessed using GenomeStudio software (version 1.9.0.24624; Illumina, CA, USA) and analyzed within the limma package [16] of the Bioconductor [17] as described elsewhere [18]: the transcription profiles were background corrected using normal-exponential model, quantile normalized and variance stabilized using base 2 logarithmic transformation.

Moderated t-test was used to detect transcripts differentially expressed between the sample groups (within limma; [16]). Storey’s q-values [19] were used to select significantly differentially transcribed genes (q < 0.05, |log_2_FC| > 1). The MIAME compliant transcription data was deposited in the ArrayExpress database (accession E-MTAB-2401).

### Real Time Polymerase Chain Reaction (RT-qPCR)

Total RNA was isolated as described in the section transcriptome analysis. Reverse transcription was performed by QuantiTeck® Reverse Transcription Kit (Qiagen, MD, USA) starting with 50 ng/μl of RNA. Final cDNA was diluted five times and quantitative real-time PCR (RT-qPCR) was performed on LightCycler 2.0 System using LightCycler® 480 DNA SYBR Green I Master Mix (Roche Diagnostics GmbH, Germany). PCR reactions (10 μl) were run according to standard manufacturer’s protocol cycled 50 times. Targets (IL-8, CXCL-1, FGF-2, VEGF-A, VIM) and housekeeping genes (GAPDH, HPRT1, COX7C, TBP, RPS9) were measured under the same conditions and from the same diluted cDNA (Additional file 2: Table S2). The results were analyzed by LightCycler software and crossing point values were determined within the R environment. Every examined cell culture was analyzed in 8 replicates (2 biological, 2 RT, and 2 technical replicates). Stability measurement of housekeeping genes and normalization was carried out using the method of Vandesompele [20].

### Statistical analysis

Statistical significance of the changes in mRNA level of the target genes between different samples and the effect of experimental proteins on K14 detection *in vitro* was determined by Student’s t-test.

## Results

### Characterization of the epithelium overlaying the nodular melanoma

**Figure 1:**
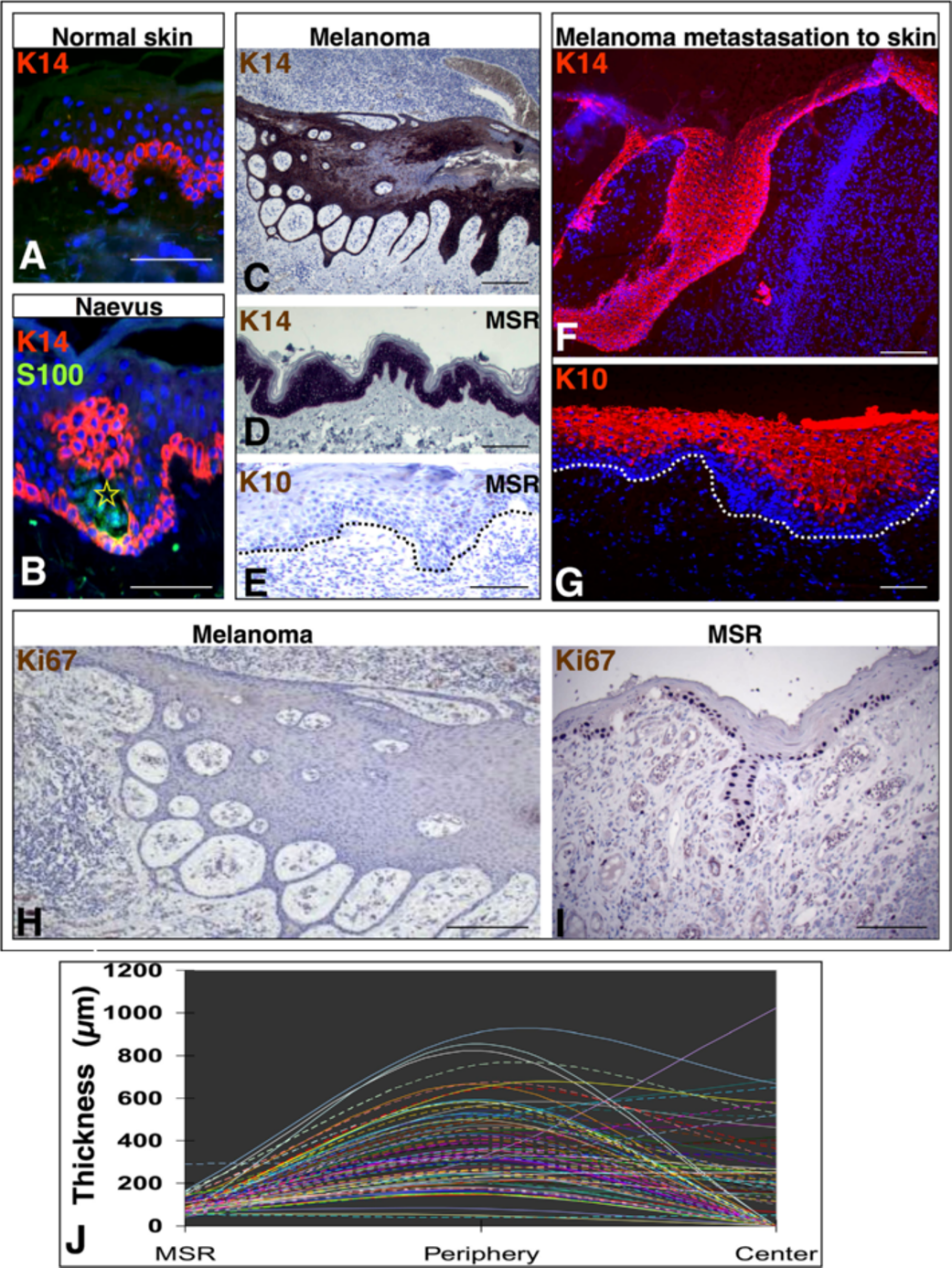
**Detection of keratin 14 (K14, A-D, F), protein S100 (B), keratin 10 (K10, E, G), and Ki67 (H, I).** K14 is expressed only in basal layer in normal epidermis **(A)**. In junctional naevi, the S100 positive naevus cells (green signal, marked by asterisk) are surrounded by K14 positive basal and suprabasal keratinocytes **(B)**. Epidermis surrounding nodular melanoma reveals pseudoepitheliomatous hyperplasia with high expression of K14 **(C)**, however, proliferation marker Ki67 is negative here **(H)**. At the margin of surgical resecate (MSR) is K14 expressed in all suprabasal layers **(D)**, this aberrant expression is accompanied by loss of differentiation marker K10 **(E)** and high proliferation revealed by the presence of Ki67 **(I)**. Similarly, epidermis overlaying dermal metastasis of melanoma is K14 positive even suprabasally **(F)**; expression of K10 is dislocated more superficially **(G)**. The epithelium in melanoma resecates reaches its thickest point on the periphery of the tumor margin **(J)**. The tumor center is covered by the epithelium of variable thickness **(J)**. Scale bars denote 25 μm.

Significantly increased thickness of the epidermis in vicinity of tumor was observed in 90% of biopsies (more than 2 times compared to epidermal thickness at the surgical margin; mean 4.56×; median 3.84; range 0.72-12.2×, p value < 0.001 by Mann–Whitney test). The increased epidermal thickness was observed on the periphery of the tumor (Figure 1 J) without significant correlation to the value of Breslow’s depth of invasion.

In the control group of normal human epidermis samples, only the keratinocytes of basal layer exhibited signal for keratin 14 (K14) (Figure 1A). Conversely, keratin 10 (K10) was here present in all suprabasal layers as expected. In junctional naevi, keratinocytes with strong expression of K14 were also observed also suprabasally in the vicinity of the melanocytic nests (Figure 1B). In case of nodular melanoma, the epidermis overlaying and surrounding the tumor exhibited various degree of hyperplasia (Figure 1C, H, J). Keratin 14 was strongly present in basal layer, but also strongly in suprabasal cells (Figure 1C). These suprabasal cells were negative for K10. Surprisingly, even the most distant epidermis at the margin of the surgical resecate also exhibited K14 positivity in the whole thickness of the epithelium along with absence of K10 signal there (Figure 1D, E). Epidermis overlaying cutaneous metastasis of melanoma demonstrated similar features, i.e., suprabasal expression of K14 (Figure 1 F) and border of K10 expression shifted more suprabasally (Figure 1G). No expression of keratin 8 (K8) and keratin 19 (K19), markers of keratinocytes with low level of differentiation, was present in epithelium overlaying primary melanoma and/or above cutaneous metastasis, except one patient (out of 20) expressing K8 (Additional file 3: Figure S1A, B). Surprisingly to the observed extent of hyperplasia, none or a very limited number of keratinocytes exhibited the marker of proliferative activity Ki67 (Figure 1H). It contrasted with a high number of Ki67-positive keratinocytes at the surgical margin of the same resecate (Figure 1I) and also to the normal control epidermis (Additional file 3: Figure S1C).

### In vitro analysis of keratinocyte-melanocyte interaction

A model of paracrine signaling during the intraepidermal melanoma development was achieved by co-culture of HPK in a transwell system with BLM, HPM or NCSC cells respectively. Characteristic markers of BLM, HPM, and NCSC cells were visualized using immunofluorescence in Additional file 4: Figure S2. The results demonstrated that both BLM and NCSC shared characteristics contrasting to HPM. NCSC were negative for MiTF that was detected as a very weak specific nuclear (and nonspecific cytoplasmic) signal in BLM and HPM.

**Figure 2:**
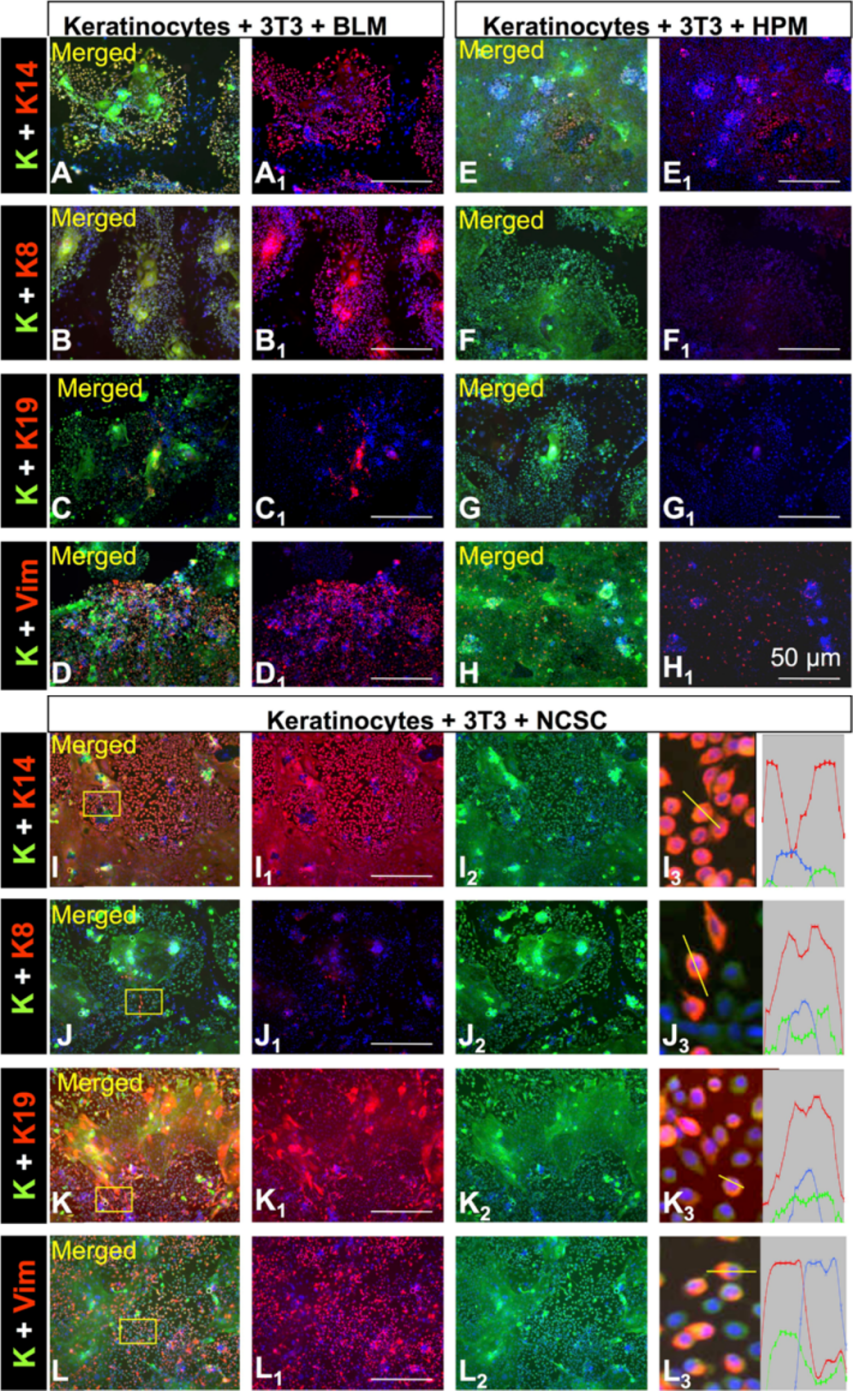
**Detection of a panel of keratins (green signal, A-L, I**_**2**_ **-L**_**2**_ **), keratin 14 (red signal, A, A**_**1**_ **, E, E**_**1**_ **, I, I**_**1**_ **, I**_**3**_ **), keratin 8 (red signal, B, B**_**1**_ **, F, F**_**1**_ **, J, J**_**1**_ **, J**_**3**_ **), keratin 19 (red signal C, C**_**1**_ **, G, G**_**1**_ **, K, K**_**1**_ **, K**_**3**_ **) and vimentin (red signal, D, D**_**1**_ **, H, H**_**1**_ **, L, L**_**1**_ **, L**_**3**_ **) in keratinocytes cultured on 3 T3 feeder cells in presence of BLM melanoma cells (A-D, A**_**1**_ **- D**_**1**_ **), of highly pigmented neonatal melanocytes (HPM, E-H, E**_**1**_ **-H**_**1**_ **) and of neural crest stem cells (NCSC, I-L, I**_**1–3**_ **-L**_**1–3**_ **).** The co-localization of signal has been verified by the measurement of fluorescence profile (I_3_-L_3_). The figures show that BLM and NCSC have a similar stimulatory effect on the expression of all the studied markers. Scale bar denotes 50 μm.

**Figure 3:**
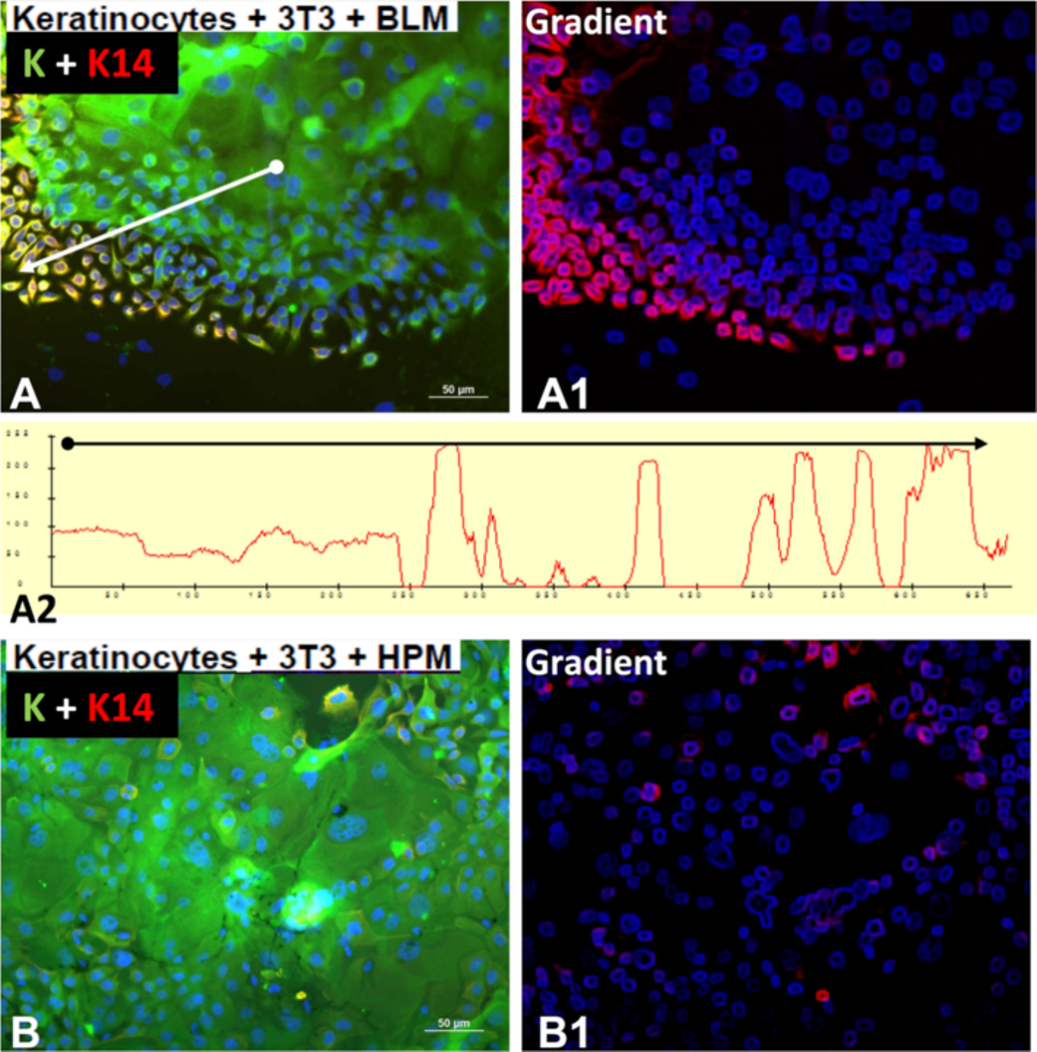
**Keratinocytes co-cultured with BLM cells exhibit accumulation of keratin 14-positive cells (A, K14, red signal) on the periphery of large colonies exhibiting keratins (green signal).** This observation contrasts with the finding in co-cultures of keratinocytes with HPM cells **(B)**. The measurements of the gradients of expression of K14 in both experimental situations **(A1, B1)** correlate with this observation. In case of keratinocytes co-cultured with BLM cells, this observation is further supported by the measurements of fluorescence intensity index **(A2)**.

The results of co-culture with melanoma cells and NCSC cells were compared to the co-culture of keratinocytes with the HPM. In co-culture with BLM cells, HPK were strongly positive for K14 and for K8 (Figure 2A, B) and in lesser extent also for K19 (Figure 2C). BLM in this system also strongly increased the number of keratinocytes co-expressing keratins and vimentin together (Figure 2D). The BLM-conditioned medium had an inhibitory effect on proliferation of human keratinocytes as documented and quantified with advantage of feeder free growth of HaCaT (Additional file 5: Figure S3). A very similar effect on HPK was also observed in co-culture with NCSC cells (Figure 2I-L). In the case of co-cultivation HPK with HPM, the expression of all studied markers was lower than in co-cultures with BLM and NCSC (Figure 2E-G).Interestingly, the expression of K14 was strong predominantly in small keratinocytes at the periphery of colonies far exceeding the intensity observed in the larger cells in the colony center (Figure 3).

Standard cultivation of keratinocytes on 3T3 feeder was performed as a control experiment (Additional file 6: Figure S4). K14-positive cells were present on periphery of keratinocyte colonies where they form an interrupted and usually narrow ring. K8- and K19-positive keratinocytes were practically absent and the number of keratinocytes exhibiting vimentin was negligible.

### Characterization of different cell types using DNA microarrays

First, we analyzed the specific influence of culture media on the transcriptome of the above-mentioned cells. As isolation and further propagation of NCSC was described exclusively in specific medium (αMEM, 10% FBS with 5% CEE), we tested initially dependence of transcription profiles of NCSC and BLM on the media without finding any major influence of the employed media (αMEM, 10% FBS with 5% CEE versus DMEM 10% FBS). This indicated possibility of running all consequent experiments in identical medium without compromising biological behavior of NCSC and BLM respectively. We also optimized cultivation of HPM in DMEM 10% FBS for this purpose.

**Figure 4:**
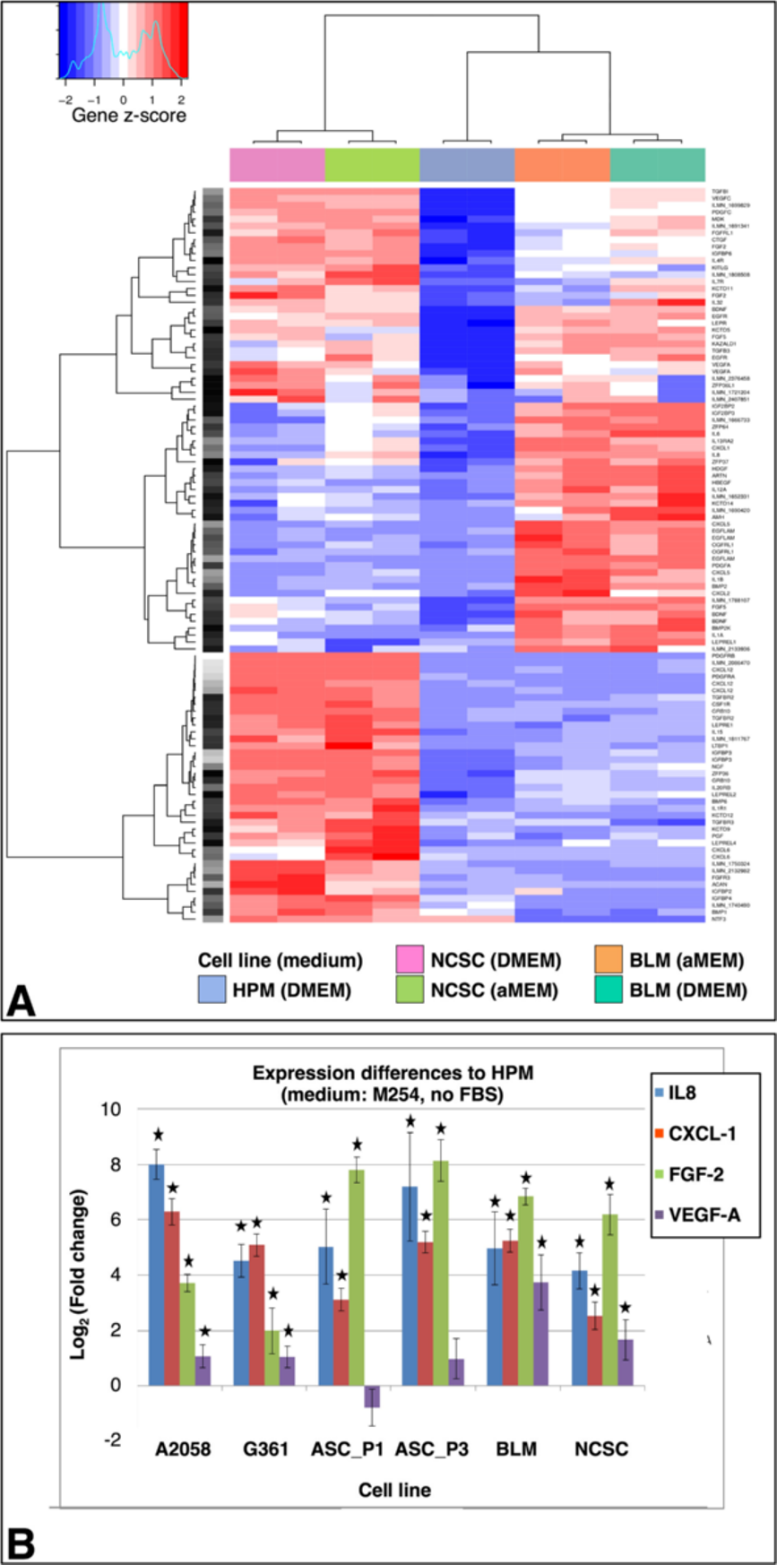
**Hierarchical clustering of transcription profiles of the genes related to extracellular factors in HPM, BLM, and NCSC (A).** Results of RT-qPCR verification of the transcriptional activity of IL-8, CXCL-1, GFG-2, and VEGF-A in BLM, NSCC, and A2058, G361 melanoma lines as well as in primary cultures of ascitic melanoma cells (Asc) after 1st and 3rd passage in relation to HPM **(B)**. Statistically significant differences from HPM at p < 0.05 significance level are marked by asterisk.

Analysis of microarrays with transcriptomes of HPM, BLM and NCSC at the whole genome scale demonstrated distinct differences among tested cell types (Figure 4A). As transcription profiles of pure cultures were well conserved in different media (Figure 4A), the biological effect in co-culture could only be attributed to particular cell types in transwells. Because this experimental setting excludes direct physical interaction of cells, we further focused our interest on extracellularly released cell products that could be responsible for paracrine regulation of the environment. In the co-culture experiments, we observed that only BLM and NCSC significantly affected the phenotype in co-cultured keratinocytes, thus we searched for the cell products that were excessively produced by these cells and not by HPM. However, the reciprocal effect of keratinocytes on pigment cell keratinocyte axis cannot be excluded. The differentially regulated factors are listed in Additional file 7: Table S3.

The expression of candidate genes whose products could participate in the control of keratinocyte phenotype (IL-8, CXCL-1, FGF-2, and VEGFA) was verified by RT-qPCR performed on lysates of cultures of the cells employed in the experiment (BLM, NCSC, and HPM). It was further extended in co-cultures using other available melanoma cell lines (A2058, G361), and primary culture of melanoma cells isolated from ascitic fluid (1st and 3rd passage was compared). Experiment with BLM, NCSC and HPM was also replicated in the medium devoid of fetal bovine serum. The results clearly demonstrated that the expression of the tested genes was upregulated in all studied melanoma cells compared to HPM (Figure 4B), with the exception of expression of VEGF-A by ascitic cells of the first passage. However, the production of VEGF-A by melanoma cells was only weakly elevated in comparison with HPM. The serum supplement to the cultivation medium had a suppressive effect on the expression of CXCL-1 in both BLM and NCSC as well as on IL-8 expression in NCSC cells (Additional file 8: Figure S5A, B).

### The role of FGF-2, VEGF-A, IL-8, CXCL-1 on cultured human keratinocytes

**Figure 5:**
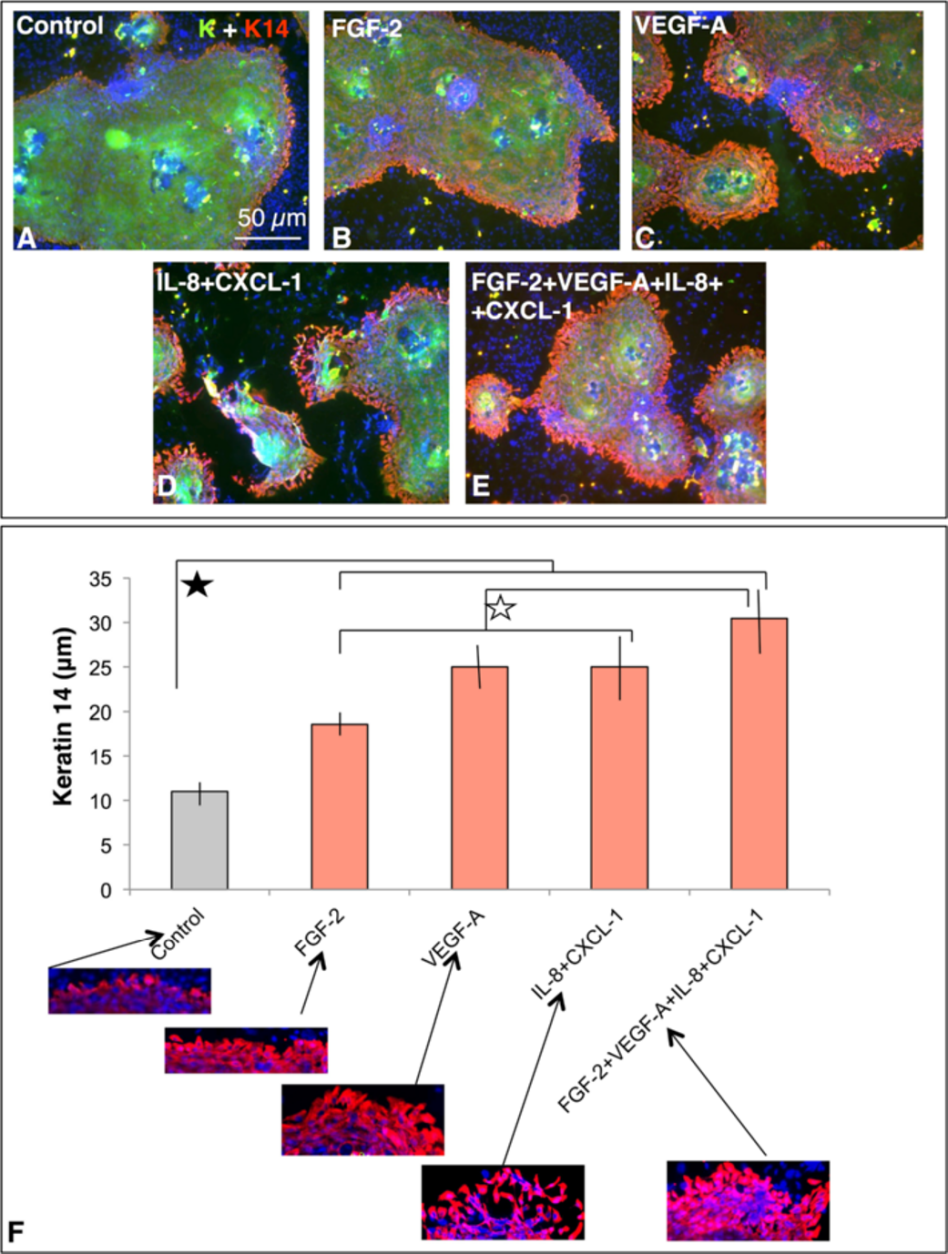
**Detection of K14 (red signal) in colonies of normal human keratinocytes (Control, A), and after the application of FGF-2 (B), VEGF-A (C), IL-8 and CXCl-1 (D), and simultaneous application of all tested substances (E).** Green signal represents keratin, bar denotes 50 μm. Panel (F) shows the difference of the thickness of K14-positive layer cells surrounding the keratinocyte colonies. All stimulated cell colonies have significantly different margin extent when compared to control colonies. The cells stimulated simultaneously by all factors have significantly wider margin than the cells treated by individual factors (p < 0.01). The peripheral cells positive for K14 are presented in higher magnification.

Keratinocytes strongly positive for K14 were located predominantly at the periphery of colonies (Figure 5A). When either FGF-2, VEGF-A and/or a mixture of IL-8 with CXCL-1 was supplemented to the culture medium, the K14-positive cells border of the colonies was significantly wider than in non-treated cultures (Figure 5B-D, F). Simultaneous addition of all tested factors to keratinocyte culture significantly increased the thickness of K14-positive edge of colony (Figure 5E, F). K14-positive cells at the border of treated colonies exhibited more elongated shape more resembling fibroblasts than usually seen in epithelial cells. These cells were not anymore in tight contact (Figure 5F).

## Discussion

This study presents morphometrical quantification of the effect of nodular melanoma on morphological appearance of surrounding epidermis, which acquires features consistent with pseudoepitheliomatous hyperplasia. We report changes in keratin expression pattern seen in the epidermal region surrounding the nodular melanoma and we have identified the candidate molecules responsible for the phenomenon. Our results correlate with reports of pseudoepitheliomatous hyperplasia in melanoma biopsies described previously [21, 22]. Others showed that, similar to K14 in this study, two connexins were induced in the epidermis adjacent to both primary melanoma and cutaneous melanoma metastases, but less so in melanocytic nevi. Also in these studies, both vertical (basal/suprabasal) and horizontal (towards the resection margin) extension of connexin expression in the epidermis adjacent to melanoma were measured. Interestingly in their studies, the extent of connexin expression in the epidermis correlated with the Breslow index of the underlying melanoma and with the proliferative index of the epidermis itself as well as partially with K6 but not K17 expression in the epidermis [9, 23].

The significance of ulceration seen in the epidermis overlaying melanoma is widely accepted and it is reflected even in the current classification system of AJCC as a predictor of worse prognosis. Existence of another biomarker, pseudoepitheliomatous hyperplasia, would be highly desirable for further improvement of melanoma patients risk stratification.

Presence of melanoma likely influences the differentiation pattern of epidermis with highly aberrant suprabasal pattern of expression of differentiation-dependent marker keratin 14 (K14), which is normally present only in basal cell layer [24]. The suprabasal expression of K14 has been reported in dysplastic epithelium [25]. This finding is further supported by simultaneous absence of keratin 10 (K10), a marker of suprabasal terminally differentiated cells in the epithelium overlaying the tumors in our study. The observed pseudoepitheliomatous hyperplastic region is also interestingly devoid of cells expressing proliferation marker Ki67. Thus, the epithelial thickening occurs even without increased rate of proliferation. This underscores depth of the change in differentiation pattern and may be the cause of the existence of hyperplastic epitheliomatous region. This hypothesis is further supported by our observation of the inhibitory effect of BLM-preconditioned media on growth of HaCaT keratinocytes *in vitro* (Additional file 5: Figure S3).

BLM melanoma cells as well as NCSC exhibit *in vitro* influence on HPK, (induced high expression of K14 and markers of very low differentiation status such as K8 or K19 [26, 27], respectively). Expression of K8 and K19 induced by BLM and NCSC was detected only *in vitro*, indicating that the process of cell dedifferentiation is more pronounced in the simplified environment of the tissue culture. This is somewhat expected as usually proliferating HPK in culture are by definition less differentiated (if not intentionally differentiated by e.g. high Calcium conditions). However, our culture systems are using Calcium concentrations exceeding 1 mM (concentration usually sufficient to induce differentiation). This further underscores the power of the intercellular interaction in our model. Moreover, the keratinocytes co-cultured with BLM or NCSC co-express vimentin along with keratins. When keratinocytes are co-cultured with normal melanocytes (HPM), such significant changes of their phenotype are not observed.

Because the keratinocytes in our co-cultures are separated from the cells in inserts by microporous membrane preventing any direct intercellular contacts, the observed changes most likely come from their paracrine activity. The transcriptomic analysis has been therefore targeted mainly on the candidate genes linked to intercellular interaction via soluble factors. Genes coding proteins such as FGF-2, VEGF-A, IL-8, and CXCL-1 have been found upregulated not only in tested BLM and NCSC but also in other melanoma cell lines (with the only exception of VEGF-A in primary culture of ascitic melanoma cells). The identified proteins are able to influence significantly the expression of K14 in keratinocyte cultures and we have also observed clear synergistic effect of these.

FGF-2 was already known as a cytokine actively produced by melanoma cells [28]. It participates on the control of dedifferentiation of normal human keratinocytes, where it stimulates expression of K14 and K19 [29]. FGF-2 is able to significantly stimulate keratinocyte migration [30]. Vimentin expression in keratinocytes co-cultured with BLM/NCSC suggests that HPK are in the stage of epithelial to mesenchymal transition, a process important for epithelial cell migration. IL-8 and CXCL-1 produced by melanoma have strong influence on progression of primary tumors [31–33]. Both these chemokines are able to stimulate significantly expression of K8 in cultured keratinocytes [15]. VEGF-A (together with IL-8) is also produced by melanomas in order to foster tumor neovascularization [34]. Keratinocytes express receptors for VEGF-A and this factor also significantly influences their biology [35].

The RT-qPCR verification of the expression of IL-8, CXCL-1, FGF-2, and VEGF-A with and without fetal bovine serum has revealed certain differences dependent on presence of the serum in the cultivation system. The serum can reduce expression of chemokines (e.g., CXCL-1) in the studied cells, more efficiently in the neural crest stem cells. This finding underscores importance of relevant study design and limited possibility of direct translation of certain *in vitro* findings without good correlation to *in vivo* situation. The results of K14 expression presented in this study demonstrate a remarkable overlap of both *in vivo* and *in vitro*. On the contrary, expression of K8 and K19 induced by BLM and NCSC was detected only *in vitro*. This finding indicates that some of the processes of cell dedifferentiation are clearly more pronounced in the simplified environment of the tissue culture. Our data also demonstrate similar biological influence of the melanoma cells and the neural crest stem cells on epidermal keratinocytes, which results from their similar transcription profiles. According to our previous study, similar change in keratinocyte phenotype occurs also in co-cultures of keratinocytes with stromal fibroblasts isolated from basal or squamous cell carcinoma or dermatofibroma [4, 5, 15, 36].

## Conclusions

**Figure 6:**
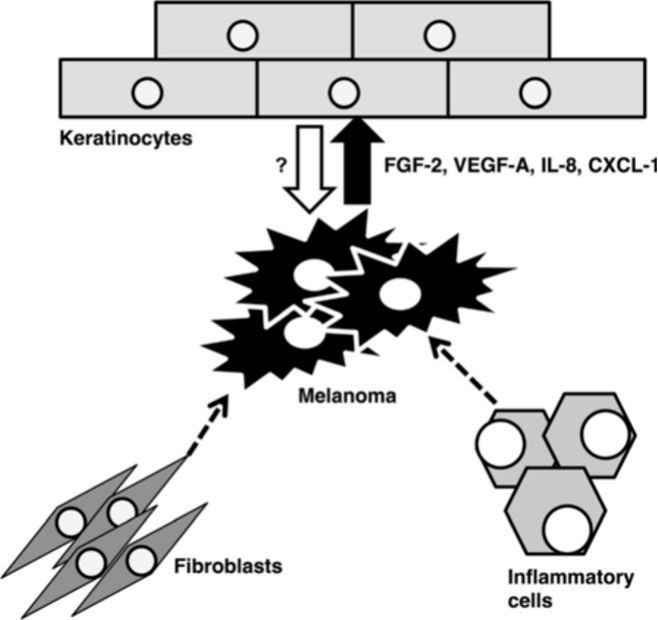
**Melanoma cell activity is in addition to other factors dependent on the activity of tumor infiltrating inflammatory cells and fibroblasts.** The melanoma cells are able to influence the differentiation pattern of keratinocytes by production of FGF-2, VEGF-A, IL-8, and CXCL-1. The reciprocal activity of keratinocytes to melanoma cells needs further research.

It should be clearly concluded here that melanoma cells are able to influence the phenotype of HPK. This observation is supported by observation of pseudoepitheliomatous hyperplasia of epidermis surrounding nodular melanoma site. Hyperplasia is accompanied by dysregulation of K14 expression in the epithelium. As summarized by Brandber and Haass [10], melanoma microenvironment includes endothelium, inflammatory cells and also keratinocytes. The keratinocytes are clearly involved in crosstalk with malignant melanocytes (Figure 6). Our data support the hypothesis of the importance of microenvironment in melanoma biology, however our understanding to these mechanisms is still limited. Mutual intercellular interactions in melanoma seem to participate in formation of this tumorigenic micromilieu. The potential role of melanoma cell-activated keratinocytes on tumor biology including metastasation shall be verified.

## Electronic supplementary material

Additional file 1: Table S1: Antibodies used in the study. (DOC 37 KB)

Additional file 2: Table S2: Primers used for quantitative real-time PCR. (XLS 30 KB)

Additional file 3: Figure S1: Detection of keratin 8 (K8, A), keratin 19 (K19, B), and Ki67 (C). K8 is expressed only in pseudohyperplastic epithelium covering nodular melanoma of 1 patient (A). K19 was negative in all studied samples (B). Only several cells of normal epidermis are positive for Ki67 (C). Scale bar denotes 25 μm. (JPEG 1 MB)

Additional file 4: Figure S2: Detection of vimentin, S100 protein, HMB45, MelanA, microphthalmia transcription factor (MiTF), and nestin in the BLM melanoma cells, the highly pigmented neonatal melanocytes (HPM), and the neural crest stem cells (NCSC). Scale bar denotes 10 μm. (JPEG 1 MB)

Additional file 5: Figure S3: Influence of preconditioned medium collected after 24 hours from subconfluent HaCaT culture (triangles) and BLM culture (squares) on proliferation of HaCaT cells. HaCaT growth in fresh un-conditioned medium (circles) is plotted for comparison Proliferation is measured by increasing confluence of the monitored cells. (JPEG 1 MB)

Additional file 6: Figure S4: Detection of a panel of keratins (K) and of keratin 14, keratin 8, keratin 19, and of vimentin in normal human keratinocytes cultured on the 3T3 feeder cells. Scale bar denotes 100 μm. (JPEG 1 MB)

Additional file 7: Table S3: Deregulated genes related to extracellular factors. The genes with statistically significant (q < 0.05) and strong (log_2_FC > 1) up-regulation in both BLM and NCSC are highlighted in green. The genes selected for downstream analyses (IL-8, CXCL-1, FGF-2, and VEGFA) are highlighted in yellow. These genes were selected according to a careful literature search and our previous studies. (XLS 47 KB)

Additional file 8: Figure S5: Results of RT-qPCR verification of the chip analysis of transcription activity of IL-8, CXCL-1, FGF-2, and VEGF-A in the cells cultured with and without 10% of FBS. The genes that are differentially expressed (p < 0.05) in BLM and NCSC, in comparison to HPM, are marked by asterisk. VIM stands for the expression of vimentin as a universal fibroblast marker. The results presented in this figure and in the Figure 3 originate from two independent experiments, which include different cell lines cultivations. (JPEG 32 KB)

## Abbreviations

AJCC: American Joint Committee on Cancer
ASC P1 and P3: Ascitic melanoma cell patient 1 and 3
CEE: Chicken embryonic extract
HPK: Human primary keratinocytes
HPM: Highly pigmented melanocytes
K14: Keratin 14
K10: Keratin 10
K8: Keratin 8
K19: Keratin 19
MC: Melanoma cells
NCSC: Neural crest stem cell.

## References

[1] Kulesa PM, Kasemeier-Kulesa JC, Teddy JM, Margaryan NV, Seftor EA, Seftor RE (2006). Reprogramming metastatic melanoma cells to assume a neural crest cell-like phenotype in an embryonic microenvironment. Proc Natl Acad Sci U S A.

[2] Hendrix MJ, Seftor EA, Seftor RE, Kasemeier-Kulesa J, Kulesa PM, Postovit LM (2007). Reprogramming metastatic tumour cells with embryonic microenvironments. Nat Rev Cancer.

[3] Plzak J, Lacina L, Chovanec M, Dvorankova B, Szabo P, Cada Z (2010). Epithelial-stromal interaction in squamous cell epithelium-derived tumors: an important new player in the control of tumor biological properties. Anticancer Res.

[4] Lacina L, Dvorankova B, Smetana K, Chovanec M, Plzak J, Tachezy R (2007). Marker profiling of normal keratinocytes identifies the stroma from squamous cell carcinoma of the oral cavity as a modulatory microenvironment in co-culture. Int J Radiat Biol.

[5] Strnad H, Lacina L, Kolar M, Cada Z, Vlcek C, Dvorankova B (2010). Head and neck squamous cancer stromal fibroblasts produce growth factors influencing phenotype of normal human keratinocytes. Histochem Cell Biol.

[6] Li L, Dragulev B, Zigrino P, Mauch C, Fox JW (2009). The invasive potential of human melanoma cell lines correlates with their ability to alter fibroblast gene expression in vitro and the stromal microenvironment in vivo. Int J Cancer.

[7] Jager MJ, Ly LV, El Filali M, Madigan MC (2011). Macrophages in uveal melanoma and in experimental ocular tumor models: Friends or foes?. Prog Retin Eye Res.

[8] Sieber-Blum M, Grim M, Hu YF, Szeder V (2004). Pluripotent neural crest stem cells in the adult hair follicle. Dev Dyn.

[9] Haass NK, Ripperger D, Wladykowski E, Dawson P, Gimotty PA, Blome C (2010). Melanoma progression exhibits a significant impact on connexin expression patterns in the epidermal tumor microenvironment. Histochem Cell Biol.

[10] Brandner JM, Haass NK (2013). Melanoma’s connections to the tumour microenvironment. Pathology.

[11] Krejci E, Grim M (2010). Isolation and characterization of neural crest stem cells from adult human hair follicles. Folia Biol.

[12] Boukamp P, Petrussevska RT, Breitkreutz D, Hornung J, Markham A, Fusenig NE (1988). Normal keratinization in a spontaneously immortalized aneuploid human keratinocyte cell line. J Cell Biol.

[13] Dvorankova B, Holikova Z, Vacik J, Konigova R, Kapounkova Z, Michalek J (2003). Reconstruction of epidermis by grafting of keratinocytes cultured on polymer support–clinical study. Int J Dermatol.

[14] Holubcova Z, Matula P, Sedlackova M, Vinarsky V, Dolezalova D, Barta T (2011). Human embryonic stem cells suffer from centrosomal amplification. Stem Cells.

[15] Kolar M, Szabo P, Dvorankova B, Lacina L, Gabius HJ, Strnad H (2012). Upregulation of IL-6, IL-8 and CXCL-1 production in dermal fibroblasts by normal/malignant epithelial cells in vitro: Immunohistochemical and transcriptomic analyses. Biol Cell.

[16] Smyth GK (2004). Linear models and empirical bayes methods for assessing differential expression in microarray experiments. Stat Appl Genet Mol Biol.

[17] Gentleman RC, Carey VJ, Bates DM, Bolstad B, Dettling M, Dudoit S (2004). Bioconductor: open software development for computational biology and bioinformatics. Genome Biol.

[18] Valach J, Fik Z, Strnad H, Chovanec M, Plzak J, Cada Z (2012). Smooth muscle actin-expressing stromal fibroblasts in head and neck squamous cell carcinoma: increased expression of galectin-1 and induction of poor prognosis factors. Int J Cancer.

[19] Storey JD, Tibshirani R (2003). Statistical significance for genomewide studies. Proc Natl Acad Sci U S A.

[20] Vandesompele J, De Preter K, Pattyn F, Poppe B, Van Roy N, De Paepe A (2002). Accurate normalization of real-time quantitative RT-PCR data by geometric averaging of multiple internal control genes. Genome Biol.

[21] Drunkenmolle E, Marsch W, Lubbe D, Helmbold P (2005). Paratumoral epidermal hyperplasia: a novel prognostic factor in thick primary melanoma of the skin?. Am J Dermatopathol.

[22] McCarty MF, Bielenberga DR, Nilssona MB, Gershenwaldb JE, Barnhillc RL, Ahearne P (2003). Epidermal hyperplasia overlying human melanoma correlates with tumour depth and angiogenesis. Melanoma Res.

[23] Haass NK, Wladykowski E, Kief S, Moll I, Brandner JM (2006). Differential induction of connexins 26 and 30 in skin tumors and their adjacent epidermis. J Histochem Cytochem.

[24] Lane EB, McLean WH (2004). Keratins and skin disorders. J Pathol.

[25] Fik Z, Valach J, Chovanec M, Mazanek J, Kodet R, Kodet O (2013). Loss of adhesion/growth-regulatory galectin-9 from squamous cell epithelium in head and neck carcinomas. J Oral Pathol Med.

[26] Michel M, Torok N, Godbout MJ, Lussier M, Gaudreau P, Royal A (1996). Keratin 19 as a biochemical marker of skin stem cells in vivo and in vitro: keratin 19 expressing cells are differentially localized in function of anatomic sites, and their number varies with donor age and culture stage. J Cell Sci.

[27] Gires O, Mack B, Rauch J, Matthias C (2006). CK8 correlates with malignancy in leukoplakia and carcinomas of the head and neck. Biochem Biophys Res Commun.

[28] Reed JA, McNutt NS, Albino AP (1994). Differential expression of basic fibroblast growth factor (bFGF) in melanocytic lesions demonstrated by *in situ* hybridization. Am J Pathol.

[29] Sun X, Fu X, Han W, Zhao Y, Liu H, Sheng Z (2011). Dedifferentiation of human terminally differentiating keratinocytes into their precursor cells induced by basic fibroblast growth factor. Biol Pharmaceut Bulletin.

[30] Sogabe Y, Abe M, Yokoyama Y, Ishikawa O (2006). Basic fibroblast growth factor stimulates human keratinocyte motility by Rac activation. Wound Repair Regen.

[31] Dhawan P, Richmond A (2002). Role of CXCL1 in tumorigenesis of melanoma. J Leukoc Biol.

[32] Payne AS, Cornelius LA (2002). The role of chemokines in melanoma tumor growth and metastasis. J Investigative Dermatol.

[33] Mangahas CR, dela Cruz GV, Friedman-Jimenez G, Jamal S (2005). Endothelin-1 induces CXCL1 and CXCL8 secretion in human melanoma cells. J Investigative Dermatol.

[34] Mashiah J, Wohl Y, Barnea Y, Schneebaum S, Gat A, Misonzhnik-Bedny F (2007). Immunohistochemical expression of platelet growth factor and vascular endothelial growth factor in patients with melanoma with and without redness (Brenner sign). Arch Dermatol.

[35] Man XY, Yang XH, Cai SQ, Yao YG, Zheng M (2006). Immunolocalization and expression of vascular endothelial growth factor receptors (VEGFRs) and neuropilins (NRPs) on keratinocytes in human epidermis. Mol Med.

[36] Kideryova L, Pytlik R, Benesova K, Vesela R, Karban J, Rychtrmocova H (2010). Endothelial cells (EC) and endothelial precursor cells (EPC) kinetics in hematological patients undergoing chemotherapy or autologous stem cell transplantation (ASCT). Hematol Oncol.

