# Supplementary material for: Melanoma cells influence the differentiation pattern of human epidermal keratinocytes
Source: Mol Cancer. 2015 Jan 5;14(1):1. doi: 10.1186/1476-4598-14-1 (PMC4325966; doi:10.1186/1476-4598-14-1)
Supplement: Supplementary file 1 — Additional file 1: Table S1: Antibodies used in the study. (DOC 37 KB) [file 12943_2014_1466_MOESM1_ESM.doc]

**Supplementary Table 1: antibodies used in study**

| **1^st^ step antibody/type** | **Supplier** | **2^nd^ step antibody** | **Chromogene** | **Supplier** |
| --- | --- | --- | --- | --- |
| MiTF/RP | Abcam, Cambridge, UK | Swine anti-rabbit | FITC | DAKO, Glostrup, Denmark |
| High molecular weight keratin/RP | Abcam |
| Protein S100/RP | DAKO |
| Thyrosinase/MM | Invitrogen, Camarillo, CA, USA | Goat anti-mouse  N-histofine Simple Stain MAX PO (MULTI) anti-mouse/anti-rabbit | TRITC or FITC  Peroxidase | Sigma-Aldrich, Prague, Czech Republic  Nichirei Biosci, Tokyo, Japan |
| Melan-A/MM | Invitrogen |
| HMB45/MM | Invitrogen |
| Nestin/MM | Abcam |
| Vimentin/MM | DAKO |
| Keratin 8/MM | DAKO |
| Keratin 10/MM | Sigma-Aldrich |
| Keratin14/MM | Sigma-Aldrich |
| Keratin 19/MM | DAKO |
| Proliferation marker Ki67/MM | DAKO |

RP rabbit polyclonal, MM mouse monoclonal
