# Supplementary figures and images for: Melanoma cells influence the differentiation pattern of human epidermal keratinocytes
Source: Mol Cancer. 2015 Jan 5;14(1):1. doi: 10.1186/1476-4598-14-1 (PMC4325966; doi:10.1186/1476-4598-14-1)

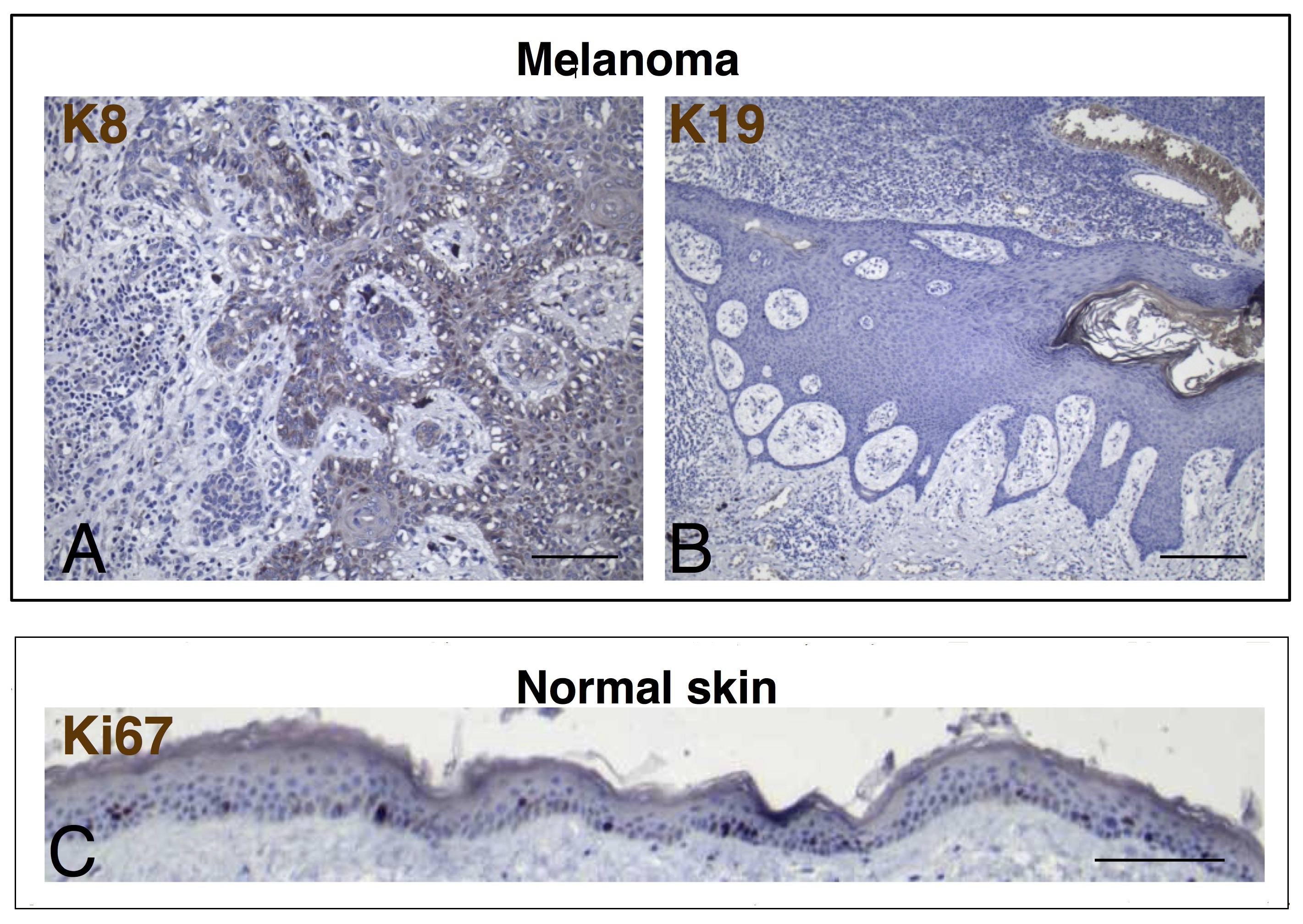

Supplement: Supplementary file 3 — Additional file 3: Figure S1: Detection of keratin 8 (K8, A), keratin 19 (K19, B), and Ki67 (C). K8 is expressed only in pseudohyperplastic epithelium covering nodular melanoma of 1 patient (A). K19 was negative in all studied samples (B). Only several cells of normal epidermis are positive for Ki67 (C). Scale bar denotes 25 μm. (JPEG 1 MB) [file 12943_2014_1466_MOESM3_ESM.jpeg]

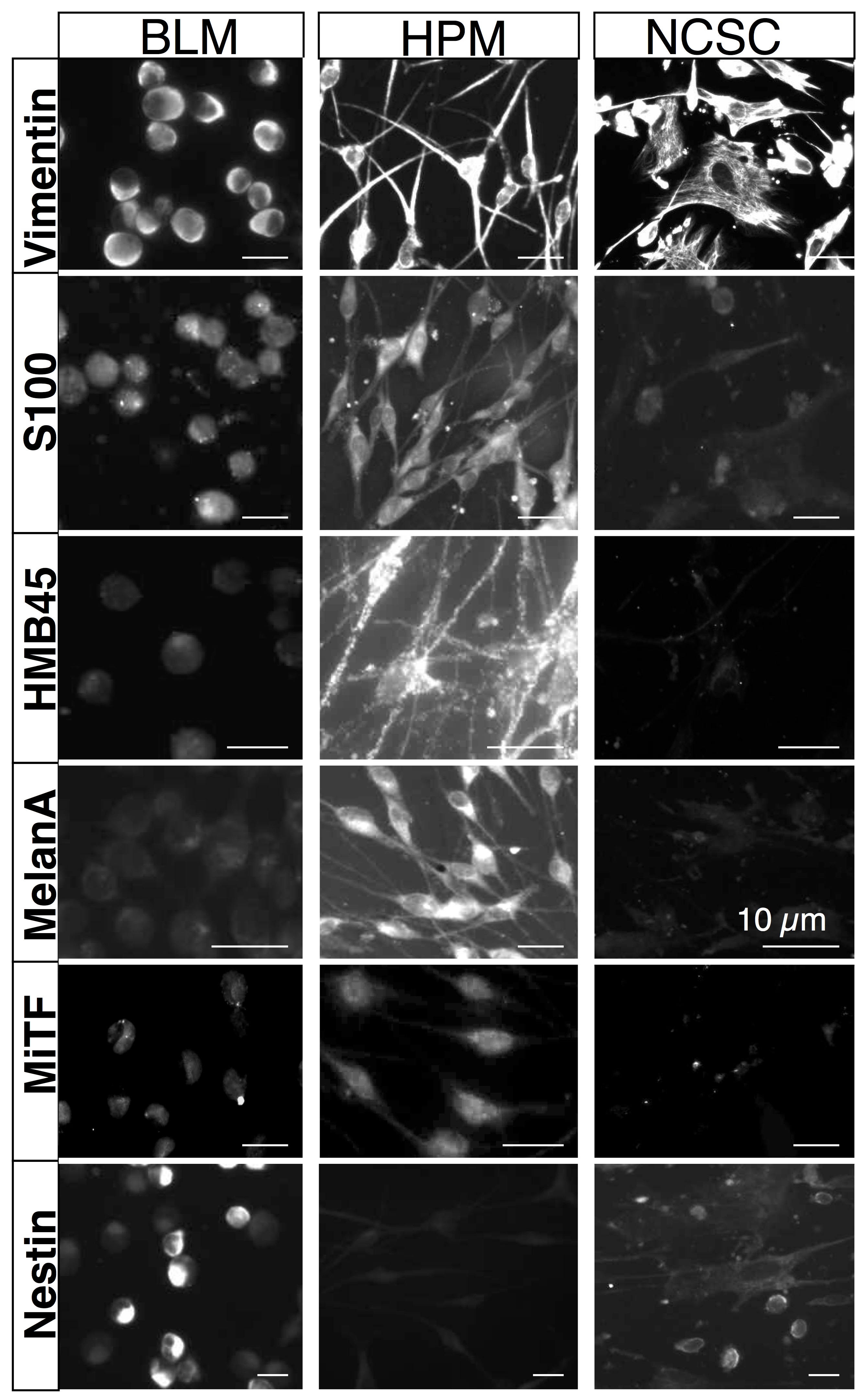

Supplement: Supplementary file 4 — Additional file 4: Figure S2: Detection of vimentin, S100 protein, HMB45, MelanA, microphthalmia transcription factor (MiTF), and nestin in the BLM melanoma cells, the highly pigmented neonatal melanocytes (HPM), and the neural crest stem cells (NCSC). Scale bar denotes 10 μm. (JPEG 1 MB) [file 12943_2014_1466_MOESM4_ESM.jpeg]

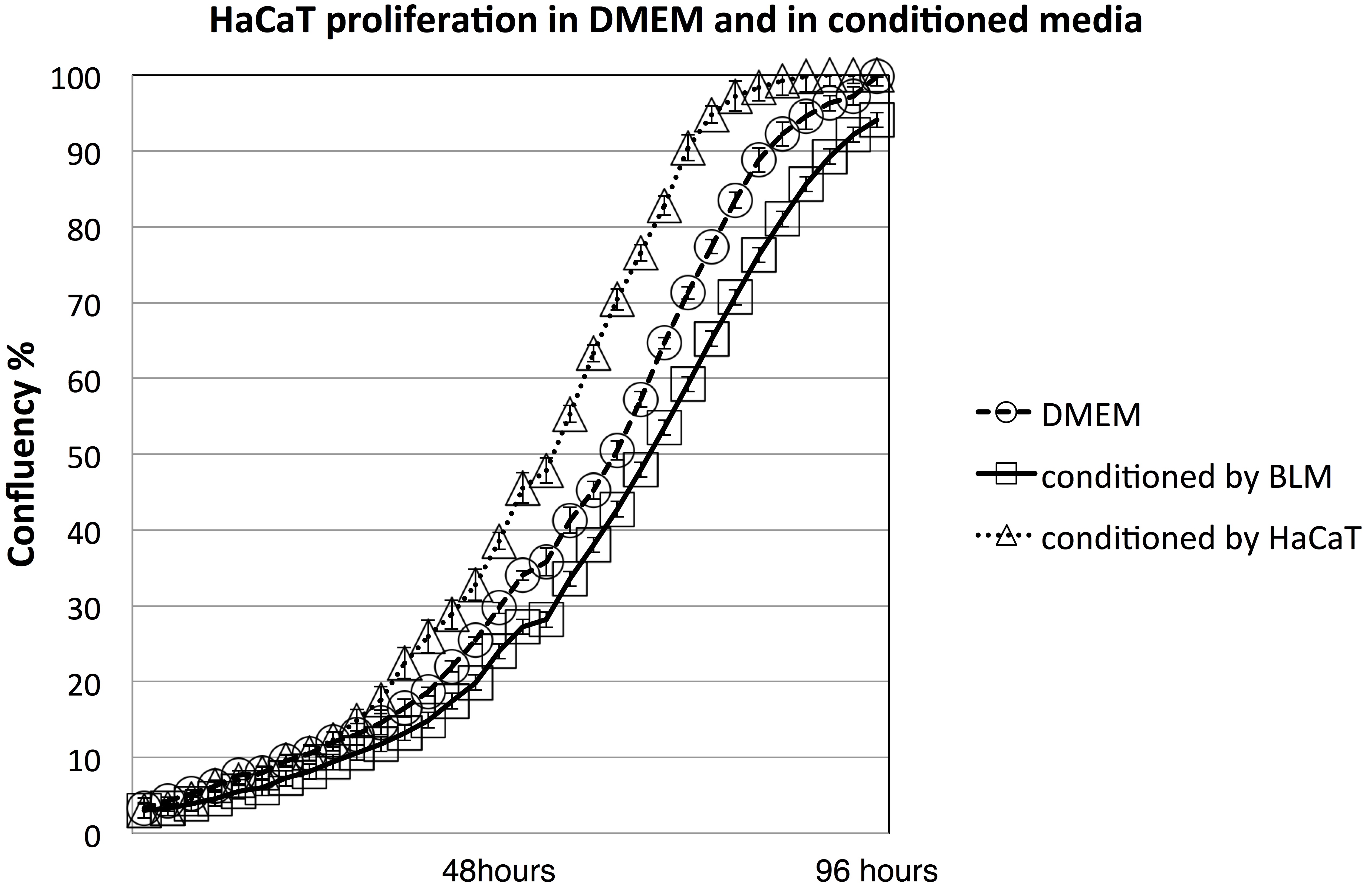

Supplement: Supplementary file 5 — Additional file 5: Figure S3: Influence of preconditioned medium collected after 24 hours from subconfluent HaCaT culture (triangles) and BLM culture (squares) on proliferation of HaCaT cells. HaCaT growth in fresh un-conditioned medium (circles) is plotted for comparison Proliferation is measured by increasing confluence of the monitored cells. (JPEG 1 MB) [file 12943_2014_1466_MOESM5_ESM.jpeg]

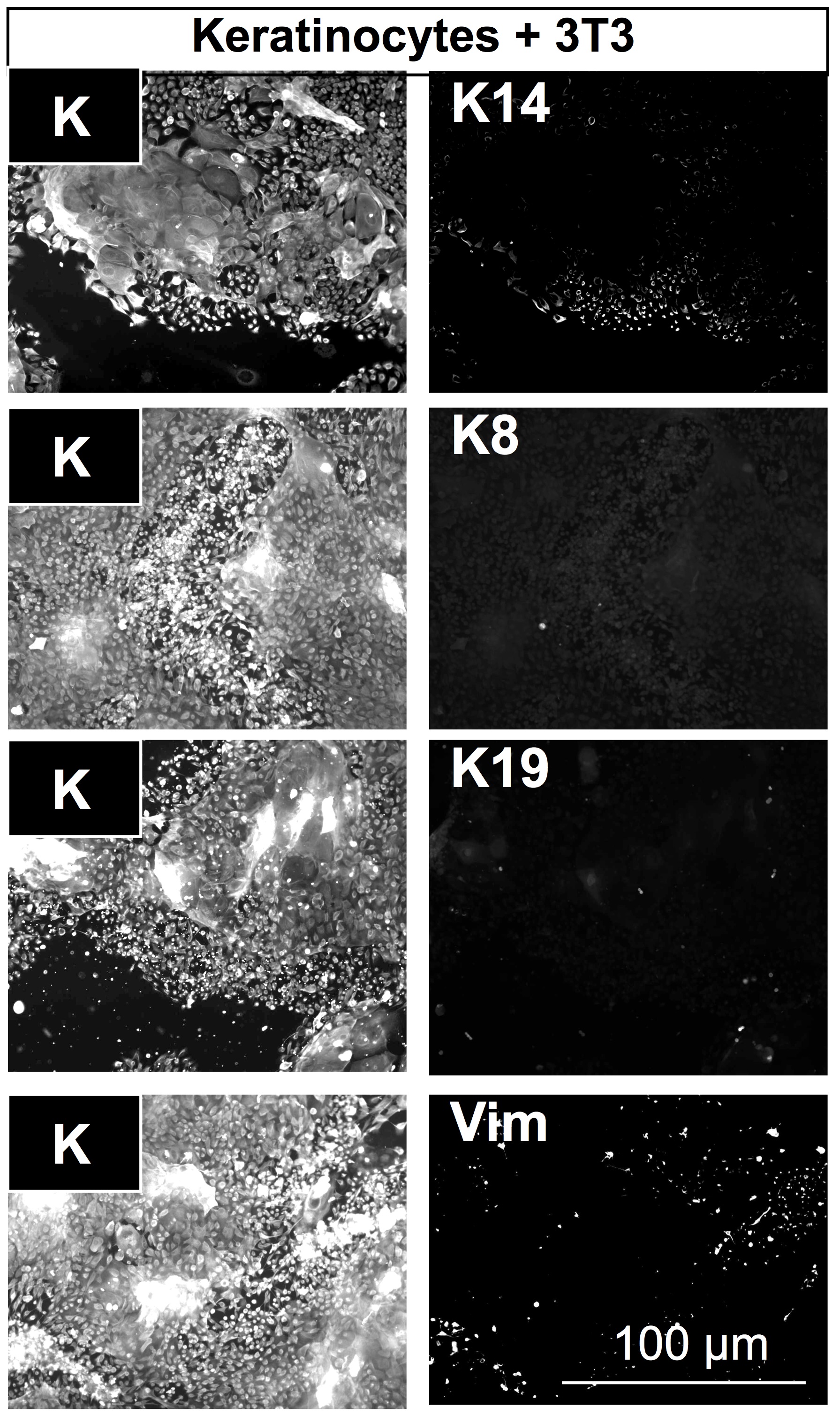

Supplement: Supplementary file 6 — Additional file 6: Figure S4: Detection of a panel of keratins (K) and of keratin 14, keratin 8, keratin 19, and of vimentin in normal human keratinocytes cultured on the 3T3 feeder cells. Scale bar denotes 100 μm. (JPEG 1 MB) [file 12943_2014_1466_MOESM6_ESM.jpeg]

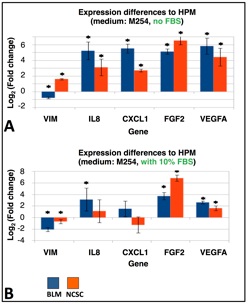

Supplement: Supplementary file 8 — Additional file 8: Figure S5: Results of RT-qPCR verification of the chip analysis of transcription activity of IL-8, CXCL-1, FGF-2, and VEGF-A in the cells cultured with and without 10% of FBS. The genes that are differentially expressed (p < 0.05) in BLM and NCSC, in comparison to HPM, are marked by asterisk. VIM stands for the expression of vimentin as a universal fibroblast marker. The results presented in this figure and in the Figure 3 originate from two independent experiments, which include different cell lines cultivations. (JPEG 32 KB) [file 12943_2014_1466_MOESM8_ESM.jpeg]
